# Role of endoscopic ultrasound-guided fine needle aspiration biopsies in diagnosing pancreatic neoplasms in the paediatric population: experience from a tertiary center and review of the literature

**DOI:** 10.2478/raon-2024-0008

**Published:** 2024-02-21

**Authors:** Maja Kebe Radulovic, Jernej Brecelj, Andrej Gruden, Margareta Strojan Flezar

**Affiliations:** Institute of Pathology, Faculty of Medicine, University of Ljubljana, Ljubljana, Slovenia.; Department of Gastroenterology, Hepatology and Nutrition, University Children’s Hospital Ljubljana, and Department of Pediatrics, Faculty of Medicine, University of Ljubljana, Ljubljana, Slovenia; University Medical Centre Ljubljana, Service of Internal Medicine, Department of Gastroenterology, Ljubljana, Slovenia.

**Keywords:** fine needle aspiration biopsy, endoscopic ultrasound, pancreatic neoplasm, paediatric pathology

## Abstract

**Background:**

Endoscopic ultrasound-guided fine needle aspiration biopsy (EUS FNAB) is a well established diagnostic method in adult patients, but is rarely used in the paediatric population. The Clinical Department of Gastroenterology at the University Clinical Centre Ljubljana and the Department of Cytopathology at the Institute of Pathology, Faculty of Medicine, University of Ljubljana, Slovenia, have been closely collaborating on EUS FNAB since the introduction in 2010. The aim of the study was to review the cases of EUS FNAB of pancreatic neoplasms in children.

**Patients and methods:**

In the digital archive of the Institute of Pathology (IP), Faculty of Medicine (FM), University of Ljubljana (UL), we found 6 cases of EUS FNAB in children, 3 had EUS FNAB of the pancreas, 2 of whom had a cytopathologic diagnosis of a tumour. In the first case, the lesion was ultrasonographically solid, and the cell sample contained branching papillary structures surrounded by aggregates of small cells with nuclear grooves. In the second case, the lesion was ultrasonographically cystic, and predominantly necrosis was seen, with only single preserved cells. Positive nuclear reaction for β-catenin was found in both cases by immunohistochemical staining.

**Results:**

In both cases, the cytopathological diagnosis of solid pseudopapillary neoplasm of the pancreas was made, the cases represent the totality of paediatric cases of pancreatic neoplasms from the Children’s Hospital Ljubljana since 2010. There were no adverse events during and after EUS FNAB. A histopathological examination of the tumour resection specimens confirmed the cytopathological diagnosis.

**Conclusions:**

Our experience indicates that EUS FNAB is a safe and effective method for diagnosing pancreatic neoplasms in the pediatric population, as supported by the findings in the literature.

## Introduction

Endoscopic ultrasonography (EUS) is a well-established diagnostic method for the evaluation of a diverse range of pancreatic lesions in adult patients, however it is a relatively new technique in paediatric pancreatology.^1,2^

Studies have also shown that EUS is a better diagnostic procedure than transabdominal ultrasound (TUS), CT scan or endoscopic retrograde cholangiopancreatography for smaller pancreatic lesions, mainly because of the proximity of the EUS probe, making possible a better evaluation of the lesion.^1,2^ EUS enables a detailed evaluation of pancreatic parenchyma and the ductal system, especially in paediatric patients with pancreatic masses, suspected autoimmune pancreatitis or fluid collections.^3,4^

In addition, it allows for the intervention procedures, namely sampling of lesions with fine-needle aspiration biopsy (FNAB) or core tissue biopsies, or performing drainage procedures in cystic lesions while using Doppler to assess and avoid the vasculature.^3^ However, the methods of cell or tissue sampling depend on the size and location of the pancreatic lesion and the professional expertise.

Pancreatic neoplasms are exceptionally rare in children, with malignant tumours having an estimated incidence of 0.02 per 100,000.^3^ It is estimated that solid pseudopapillary neoplasm (SPN) accounts for up to 71% of pancreatic tumours in children and adolescents, followed in order of frequency by pancreatic neuroendocrine tumour, serous cystadenoma and pancreatoblastoma, which is the most common malignant tumour in the patients under 10 years old.^3,5,6^

The radiologic preoperative diagnosis usually starts with a TUS, which allows a diagnosis of most paediatric pancreatic tumours, except for functional neuroendocrine tumours like insulinomas and gastrinomas, which may cause symptoms even though they’re small.^3^ This is especially true in the case of SPN, which presents as a well demarcated lesion, with cystic or necrotic components and with occasional calcifications within solid tumour.^3,4^

The Clinical Department of Gastroenterology at the University Clinical Centre Ljubljana and the Department of Cytopathology at the Institute of Pathology (IP), Faculty of Medicine (FM), University of Ljubljana (UL), Slovenia, have been closely collaborating on endoscopic ultrasound-guided fine needle aspiration biopsies (EUS FNAB) since the introduction of the method in 2010. The aim of present study was to analyse the cases of EUS FNAB of pancreatic neoplasms in children in a tertiary care centre and to incorporate a review of the existing literature.

## Patients and methods

### Study design

A retrospective institutional case series with a literature review was made to assess the outcomes of EUS FNAB in our tertiary referral centre. From January 2010 to December 2021, 6 EUS FNAB of gastrointestinal lesions were performed on paediatric patients (age < 18 years), coming from the Department of Gastroenterology, Hepatology and Nutrition (GHN) of the Children’s Hospital Ljubljana. The study was approved by Review Board of the Institute of Pathology UL FM (ID 4/23).

### Endoscopic ultrasound-guided fine needle aspiration biopsy

EUS FNAB procedures were done by experienced gastroenterologists specialized in endosonography and US guided FNAB. Three children had EUS FNAB of the pancreas because of a TUS detected tumour: in one, only normal pancreatic tissue was retrieved, with no tumour on follow-up, the other two had a tumour. A primary cytopathological diagnosis in both cases was that of a solid pseudopapillary neoplasm (SPN) (Table 1). According to the search of the hospital and institute’s databases, these were also the only cases of pancreatic neoplasms diagnosed at GHN.

**TABLE 1.** Results of the immunochemical reactions in both cases, on cytological and histological samples

**Case**	**β-Catenin**	**Cyclin D1**	**Synapto physin**	**Chromo granin**	**CD56**	**PR**	**CD10**	**CKAE1/AE3**	**SOX11**
**1 cyto**	**+**	**+**	**−+**	**−**	**+**	**/**	**+**	**−**	**+**
**1 histo**	**+**	**/**	**/**	**/**	**/**	**/**	**/**	**/**	**+**
**2 cyto**	**+**	**+**	**+−**	**−**	**−+**	**−**	**+**	**−+**	**+**
**2 histo**	**+**	**+**	**Focally +**	**/**	**/**	**/**	**/**	**/**	**+**

Cyto = cytology samples obtained by endoscopic ultrasound-guided fine needle aspiration biopsy (EUS FNAB); Histo: histology samples from the tumour resection specimen

+ = positive immunostaining reaction; − = negative immunostaining reaction; / = immunostaining was not performed; +− = immunostaining reaction predominantly positive; −+ = immunostaining reaction predominanty negative

In all the cases written informed consent from the parents was obtained for the procedure. The procedures were done in general anaesthesia with a linear probe echoendoscope (EG-580UT, Fujifilm, Japan). FNAB was performed with a 22-gauge Boston Acquire FNAB needle (Boston Scientific, Marlborough, MA, USA).

In all the cases, a cytopathologist was present for a rapid on-site evaluation (ROSE) of the sample cellularity. For ROSE, one direct smear was prepared at the patient’s side, fixed in Delaunay fixative (absolute ethanol:acetone 1:1 with 0.05 % 0.5M trichloroacetic acid) and stained with Hemacolor for immediate cytomorphological examination (and later Papanicolaou stained in the laboratory). The second direct smear was air-dried (for later Giemsa staining). The remaining sample was rinsed and stored in an in-house made cell medium.^7^ There was a maximum of 5 passes in one case with initial unsatisfactory samples (e.g. samples without diagnostic cells). Subsequently, all the samples were sent to the cytopathology laboratory for further preparation. If necessary, the samples were filtered to remove blood, concentrated or diluted to obtain uniform monolayers of diagnostic cells by subsequent cytocentrifugation (Shandon Cytospin 4, ThermoScientific, UK). In both cases with a neoplasm, tissue fragments were present and cell blocks were prepared by transferring clots and/or any visible tissue fragments into standard tissue cassettes. Samples were further fixed in formalin and then processed according to the standard procedures for tissue samples. After the cytopathological examination of the slides, the immunocytochemical staining was conducted using the automated immunostaining system ULTRA by Ventana Medical Systems Inc., Tucson, Arizona, USA, on the cytospins and formalin-fixed, paraffin embedded tissue sections from the cell blocks. The detection of bound primary antibodies was carried out with the optiView detection kit, except for CD 10 and NSE which were detected by iView, all from the same company.

The antibody panels utilised on cytospins consisted of following markers:

Vimentin (Recombinant Anti-Vimentin antibody, clone EPR3776, Cytoskeleton Marker, ABCAM), CD56 (Rabbit monoclonal antibody, Clone MRQ-42, Cell Marque), CD10 (clone 56C6) and CKAE1/AE3 from the same producer (Leica Biosystems), NSE (Monoclonal mouse anti-human neuron specific enolase, clone BBS) and Ki67 (Monoclonal mouse antihuman antigen, clone MIB-1) from the same producer (Agilent Technologies), chromogranin (anti-chromogranin A, primary antibody, clone LK2H10) and synaptophysin (Rabbit monoclonal antibody, clone MRQ-40) and PR (Anti-progesterone receptor, Rabbit monoclonal primary antibody, clone 1E2) and CyclinD (Anti-Cyclin D1, Rabbit monoclonal primary antibody, clone SP4-R), all from Ventana Medical Systems Inc.

Reactions with additional antibodies were performed on the cell block sections using following reagents: E-Cadherin (Mouse monoclonal antibody, clone NCH-38, Agilent Technologies), β-catenin (Mouse monoclonal antibody, clone 14) and SOX11 (Mouse monoclonal antibody, clone MRQ-58), all from the same producer (Cell Marque).

For our literature review, we conducted a comprehensive search on PubMed to identify cases of SPN diagnosed through EUS-FNAB. We employed the following key terms during the search process: ‘EUS-FNAB (in full) and pediatric patients’ and ‘EUS-FNAB (in full) and solid pseudopapillary neoplasia.’ Given the rarity of pediatric SPN cases, we opted to include all relevant results, ranging from research articles to case reports. The gathered information has been compiled in Table 2 for a thorough examination of the available data.

**TABLE 2.** Pancreatic neoplasms diagnosed by FNAB in the pediatric population: cases in the literature

**Study**	**No of cases/No with tumour**	**Sex/age (y)**	**EUS-FNAB non tumour diagnosis**	**EUS-FNAB tumour diagnosis**	**EUS-FNAB complications**	**Tumour histology**	**Follow-up**
Nabi Z *et al.*^8^	34/23	NA/Median age 15 (8–18)	Inflammatory mass, Pseudocyst, Lymphoepithelial cyst, Epithelial cyst	SPN (21), Pancreatoblastoma (1), Round cell tumor (1)	Throat pain (7), abdominal pain (2), self-limiting bleeding (2), fever (1)	88% confirmed EUS-FNA diagnosis	NA
Al Rashdan A *et al*.^9^	9/3	NA/Median age 16 (4–18)	Cysts, Inflammation	SPN (2), Carcinoid tumour (1)	None	SPN	Uneventful (SPN), Died of metastatic disease (Carcinoid tumour)
Gordon K *et al*.^10^	6/3	NA/Average weight 70 kg	Pseudocyst (4), multiple unilocular cysts (1)	SPN (2), Insulinoma	Mild pancreatitis (1)	NA	Multiple endocrine neoplasia 1 (Insulinoma)
Jia Y *et al*.^11^	1/0	F/13	Simple pancreatic cyst	None	None	None	None
Mahida JB *et al*.^12^	1/1	F/13	None	SPN (1)	None	SPN	No recurrence
Attila T *et al*.^13^	6/2(3)*	4M,2F/10-16	Focal pancreatitis, Chronic pancreatitis	B-cell lymphoma, Islet cell tumour, Suspicious of malignancy	None	B-cell lymphoma, Islet cell tumour, Sclerosing pancreatitis	Multiple endocrine neoplasia 1 (1), NA
Bardales RH *et al*.^14^	2/2	F/13, 18	None	SPN (2)	NA	SPN	None
Nadler EP *et al*.^15^	1/1	F/13	None	SPN (1)	None	SPN	No recurrence.

EUS FNAB = endoscopic ultrasound-guided fine needle aspiration biopsy; F = femaleM = male; NA = not available; SPN = solid pseudopapillary neoplasm

## Results

The first case was a 8-year-old female patient, weighting 40 kg, who was referred to EUS FNAB from GHN due to a dense lesion in the pancreas found by TUS when searching for the cause of increased liver function tests (AST 1.19 µkat/L [normal up to 0.52 µkat/L], ALT 1.19 µkat/L [normal up to 0.52 µkat/L], normal gama-GT, alcaline phosphatase and bilirubin) performed due to nonspecific disease signs (fatigue, nausea, headache).

**FIGURE 1.**
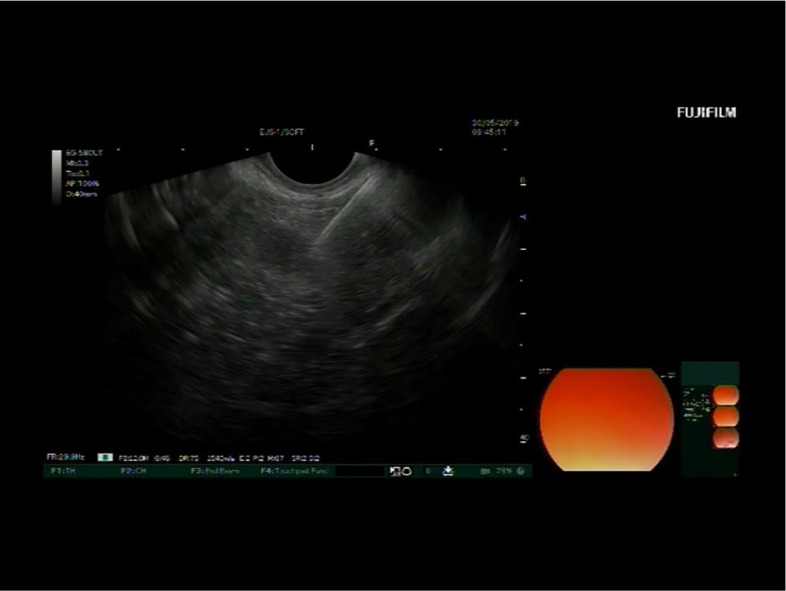
Case 1 during endoscopic ultrasound-guided fine needle aspiration biopsy (EUS FNAB). Linear probe echoendoscope localised in duodenum showing a round, well circumscribed, isoechogenic lesion in the pancreatic neck that was punctured transduodenaly. Tip of the needle (upper right) is in the lesion.

**FIGURE 2.**
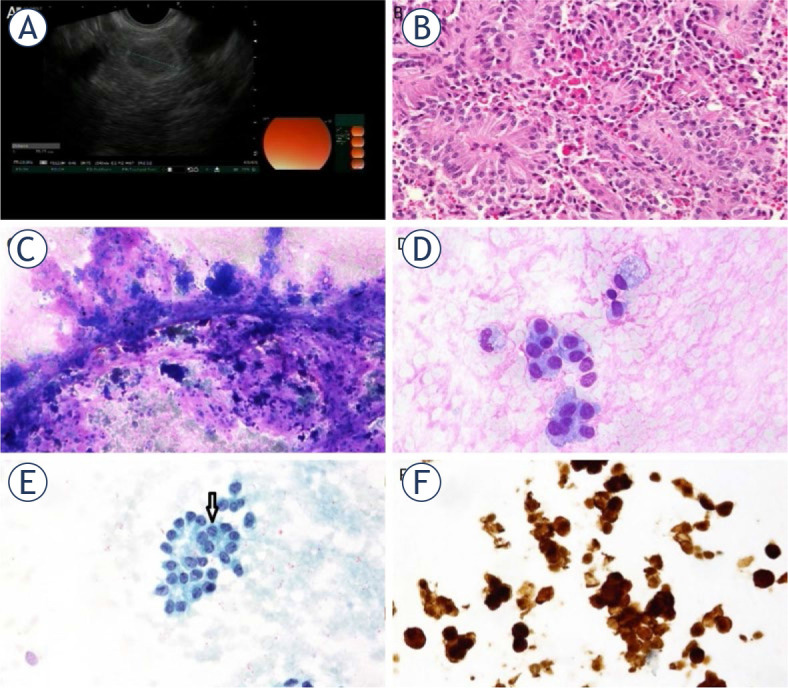
Case 1 **(A)** Endoscopic ultrasound (EUS). A well demarcated, isoechogenic tumour with more echogenic thin border, probably representing a solid pseudopapillary neoplasm (SPN). **(B)** Pseudopapillary structures in a resected specimen (HE, X200). **(C)** Endoscopic ultrasound-guided fine needle aspiration biopsy (EUS FNAB). Branching pseudopapillary structures with cohesive cellular clusters and a pinkish substance in the background (MGG, X100). **(D)** EUS FNAB. Small clusters of bland cells with a foamy macrophage and eosinophilic substance in the background (MGG, X400). **(E)** Cytospin showing a group of monomorphous cells with round/oval nuclei with smooth, indented nuclear membrane and grooves (arrow) (Pap, X400). **(F)** Positive nuclear β-Catenin reaction in the cell block (X400).

EUS showed a round, well circumscribed, isoechogenic lesion in the pancreatic neck with a diameter of 15 mm (Figures 1, 2A). The cell sample was highly cellular, with abundant eosinophilic stroma, around which the tumour cells were arranged in rounded clusters (Figure 2B–C). The individual cells had moderate, basophilic cytoplasm. The nuclei were round to elongated, with grooves, the chromatin was pale with incospicuous nucleoli (Figure 2D–E). The background of the specimen contained blood and a few siderophages. Only a few tumour cell nuclei were positive for the proliferation marker Ki67 (less than 1%), the rest of the immunocytochemical reactions were consistent with the final cytopathological diagnosis of SPN (Table 1) (Figure 2F). Additional immunohistochemical reactions revealed positive results for Vimentin, CD56, CD10, and NSE, while E-Kadherin showed a negative result.

Eighteen days after EUS FNAB, a partial resection of the pancreas with the lymph node dissection at a. hepatica communis and cholecystectomy followed. The frozen section and definitive specimens showed a solid pseudopapillary tumour confined to the pancreas, measuring 18×10 mm in the largest diameter, lobulated, homogeneous, removed 3 mm from the pancreatic surgical margin (Figure 2B). There was no perineural or lymphovascular invasion, and no tumour tissue was present in the resected lymph node. The patient reported no problems at the outpatient follow-up, the weight gain was adequate, postoperative US showed no additional abnormalities and a fecal elastase, which assesses exocrine pancreatic function, was normal. Thirty-nine months after the EUS FNAB she had acute pancreatitis, which resolved with conservative treatment. Magnetic resonance imaging of the pancreas and abdominal MR with contrast showed no recurrence. At the last follow-up 50 months after the initial diagnosis, she had no medical complaints.

**FIGURE 3.**
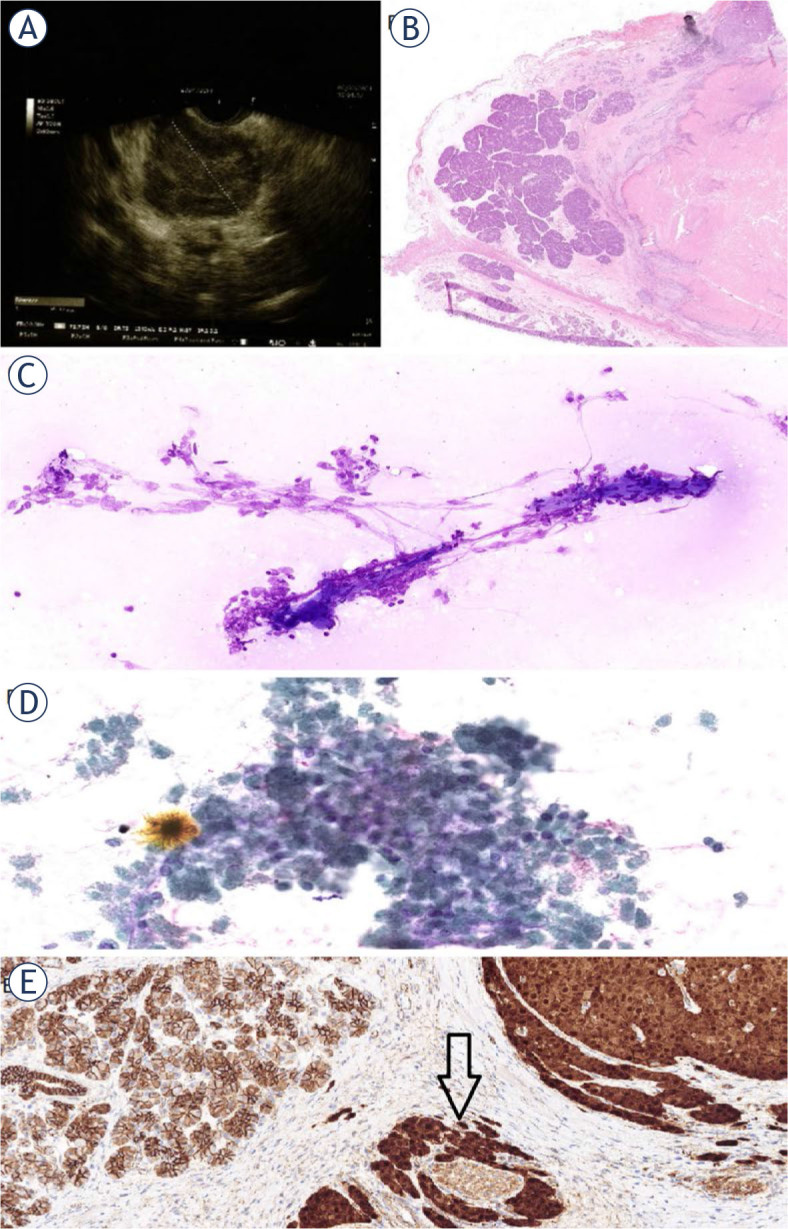
Case 2 **(A)** Endoscopic ultrasound (EUS). A well demarcated, hypoechogenic tumour. **(B)** A slide of a resected pancreas at low magnification showing an area of normal pancreatic tissue (left) and an area of eosinophilic degenerative necrosis (right) (HE, X3). **(C)** Endoscopic ultrasound-guided fine needle aspiration biopsy (EUS FNAB). Naked capillaries surrounded by necrotic cells and a group of cells with preserved nuclei (MGG X100). (**D**) EUS FNAB. A group of mainly necrotic cells and a few cells with preserved nuclei in between. **(E)** A slide of a resected pancreas at high magnification as in **(B)** with a positive nuclear β-Catenin reaction in the tumour (right) with a perineural invasion (arrow) (X100).

The second case was a 7-year-old female patient, weighting 43 kg, who came to EUS FNAB from the GHN where she presented with abdominal pain. A pancreatic cystic lesion was noted on TUS exam, which could represent a tumour or pseudocyst. EUS showed a 40×40 mm hypoechogenic tumour in the head of the pancreas, pressing the portal system and causing dilatation of the ductus choledochus to 9 mm (Figure 3A). On ROSE the EUS FNAB samples were all non-diagnostic (only blood), except for one pass, where numerous lymphocytes, histiocytes and a necrotic background was retrieved. In the filtered and concentrated sample, there were few preserved tumour cells. They were monomorphus, small, with a round, regular nuclei, single nucleoli and focally spickled chromatin (Figure 3D). The cytoplasm was coarsely granular. In the background, poorly preserved branched capillaries, surrounded by predominantly necrotic cells were visible (Figure 3C). According to the immunochemical reactions in the rare preserved cells, the cytopathologic diagnosis was SPN (Table 1). Thirteen days after EUS FNAB, a partial resection of the pancreas with duodenum, excision of the lymph node adjacent to the a. hepatica communis and cholecystectomy followed. Histopathologic examination confirmed SPN with extensive necrosis of the pancreatic head, with the largest diameter of 30 mm, at a distance of 0.1 mm from the nearest retropancreatic surgical margin (Figure 3B). There were perineural as well as lymphatic and vascular invasions (Figure 3E). No involvement with tumour cells was present in the excised lymph node and 6 regional lymph nodes. The gallbladder and duodenum were un-remarkable. Given the extensive tumour necrosis and lymphovascular invasion, the report mentioned the possibility of a more aggressive clinical course. Postoperative follow-up was uneventful. TUS showed changes consistent with the post-operation state. The stool elastase was reduced. Despite the absence of clinical signs, pancreatic enzyme therapy was initiated to ensure adequate nutrient resorption. She has gained considerable weight and has grown appropriately. The girl was presented to the haemato-oncology multidisciplinary team, which advised MRI of the abdomen every 3 months for the first 2 years and then every 6 months for up to 5 years, which she regularly undergoes. At the last follow-up, 31 months after the initial diagnosis, she had no complaints, the laboratory results and the abdomen MRI showed no signs of disease recurrence.

The third case, which ultimately did not involve a pancreatic tumor, pertained to a 17-year-old boy with trisomy 21, weighing 46 kg. He exhibited an enlarged and inhomogeneous pancreatic head on TUS and magnetic resonance cholangiopancreatography following treatment for biliary pancreatitis that resolved without the need for endoscopic retrograde cholangiopancreatography. EUS-FNAB revealed no tumor cells. Consequently, a pancreatic biopsy was recommended, but the patient’s mother declined. Subsequent TUS follow-ups showed no evidence of tumor growth or disease progression.

Given the patient’s concurrent mild exocrine pancreatic insufficiency (faecal elastase 88 µg/g, normal > 200 µg/g), pancreatic enzyme replacement therapy was initiated. However, a more comprehensive diagnosis of the pancreatic disease, such as autoimmune or hereditary pancreatitis, proved unattainable as the family withheld consent, and the patient failed to attend follow-up visits.

## Discussion

The use of EUS FNAB in the paediatric population is limited, even though it is a well-established diagnostic procedure for the assessment of suspicious pancreatic tumours in adult patients. In paediatric cases, the primary indication for EUS-guided pancreatic tissue sampling is pancreatic mass or suspected autoimmune pancreatitis. These conditions often manifest as inhomogeneous lesions of the pancreas, detectable through TUS or EUS.^3^ Our retrospective study spanned the whole period since the introduction of EUS FNAB in the tertiary heath care centre in 2010 until the end of the review period in December 2021. During that period, we had the first case of EUS FNAB in a child in 2019 followed by 5 more cases with complete cytopathological reports and corresponding archival slides. Among the total of 6 children with EUS FNAB of gastrointestinal lesions, two had pancreatic neoplasm, diagnosed as SPN by cytopathological examination.

According to the reviewed literature (Table 2), the adoption of EUS for children with pancreatobilliary diseases was delayed due to different reasons, among them the low incidence of tumorous lesions and even rarer malignancies.^8^ Other important reasons were also the size limitation of EUS equipment relative to pediatric anatomy, the low number of skilled pediatric endoscopists with EUS expertise, the need for sedation or general anesthesia, and a limited awareness among pediatric practitioners of EUS diagnostic and therapeutic possibilities.^3,8^ In our patients, the procedure and equipment used was similar to the adult population, apart from the use of general anaesthesia. The endoscopists were very experienced in the management of adult patients, enabling the procedure to be safe and efficient also in the pediatric patients, which corresponds well with other studies.^1,3,8,9,10,11,16,17,18^ While EUS was shown previously as a safe diagnostic method in the pediatric population, covering the age range of our patients (age 8 and 9 years, respectively), there is limited experience with EUS especially in smaller children (< 15 kg) due to the fear of an esophageal rupture associated with the large diameter (adult) EUS scope. Therefore, for smaller children (< 15 kg) endobronchial ultrasound has been used instead and a few studies have shown that EUS can be performed safely and with a high diagnostic accuracy even in these children.^1,3,8,9,10,11,16,17,18^

The initial diagnostic radiologic assessment of pancreatic lesions is usually conducted by a TUS, which was also the approach in our two paediatric patients. In one patient, a solid isoechogenic lesion suspicious for tumour was found in the neck of the pancreas. For radiologically solid lesions, the differential diagnoses include autoimmune pancreatitis, neuroendocrine tumours, microcystic serous adenoma, pancreatoblastoma and acinar cell carcinoma.^19^ Autoimmune pancreatitis can also closely mimic radiologic impression of a tumour, so tissue acquisition for light microscopic morphological evaluation is a crucial step in reaching a diagnosis.^1,3,16^ However, a cytologic impression can also be false positive.^13^

In the second patient, a pancreatic cystic lesion was noted on TUS, which was interpreted as either a tumor or a pseudocyst. Cystic lesions of the pancreas may represent benign or malignant processes and are identified incidentally during abdominal cross-sectional imaging or TUS performed for other indications.^3,8^ The main radiologic differential diagnoses in the case of predominantly cystic lesions in children are pseudocysts after a blunt pancreatic injury, retention cysts in cystic fibrosis patients, congenital anomalies (duplication cyst), a part of syndromes like von Hippel Lindau or a tumour pathology of a cystic appearance, such as SPN, neuroendocrine tumours, macrocystic serous adenoma, or mucinous cystic neoplasm.^3,8,19^

SPN is a low-grade tumour that mainly affects younger women and, to a lesser extent, children. It accounts for up to 30% of pancreatic tumours in patients under 40 years of age.^20^

It may be clinically silent as in case 1 or presents as an abdominal pain for its mass effect as in case 2, or rarely as jaundice.^20^ Usually it does not have an endocrine function and the tumorous markers are negative.^20^

The radiologic imaging in our cases covered the ends of the spectrum of the adult SPN presentation which is illustrated by its old name of “solid-cystic tumour” reflecting its radiological picture of a solid tumor with necrotic cystic areas, which are more common in larger tumours.^20^ However, we present a pediatric case of one completely solid and one almost completely cystic lesion, without the predominant solid-cystic appearance. Another difference was also the location of the lesions, with one located in the pancreatic head and one in the pancreatic neck, as opposed to the usual body or tail presentation.^20^

The demographics matched the incidence of pediatric SPN with exclusively female patients, the youngest being 8 years old, as is also the case in the literature.^20^ Nevertheless there are anecdotical cases of tumors in pediatric males.^21,22,23^

With EUS one gains the ability to better characterise pancreatic cystic and less common solid lesions also in children and, when performed in combination with FNAB, the discrimination between the various types of cysts and solid tumors can be made, that my direct treatment from a surgical resection to chemotherapy.^13^ In our cases, ROSE was used to assure diagnostic cell samples and a specific diagnosis of SPN could be made in both cases based on cytomorphology supplemented by relevant immunocytochemical stainings.

The morphological and immunocytochemical features of SPN in paediatric population don’t differ from adult population. In the cytopathological specimens, SPN comprises pseudopapillary clusters, acinar groups and single cells, delicate capillaries and globules of amorphous myxoid material which was well presented in case 1, but not in case 2 which was mainly necrotic.^24–25^ Histomorphologically and cytomorphologically, the tumour is most commonly composed of unimorphic cells, with sparse to moderately abundant cytoplasm, which may be pale or oncocytic, with vacuoles and round to oval nuclei, with grooves and a fine chromatin structure, which also presented in our cases.^24–25^ There is however an exception, where a few large pleomorphic atypical multinucleated giant cells could be intermingled with more typical ones, which was not found in our cases.^25^

Main cytopathologic differential diagnosis of SPN in children depends on the age, comprising neuroendocrine neoplasms in patients older than 10 years and a pancreatoblastoma in younger patients.^3^ In comparison to neuroendocrine neoplasms, SPNs have hyaline-mucinous stroma, cytoplasmic vacuoles, nuclei with grooves and negative reactions to chromogranin and cytokeratin.^20,26,27^ Pancreatoblastoma, which occurs in younger children (median age 5 years) combines several types of differentiation, namely acinar, endocrine and ductal with morulae, with corresponding immunoprofile. Cells are positive for cytokeratin AE1/AE3, BCL 10, neuroendocrine markers, EMA, and have PAS-D positive granules in the cytoplasm.^20,26,27^

For the concordant final cytopathological diagnosis of SPN, a combination of cytomorphological and immunohistochemical findings is widely recommended.^26^ To differentiate between neuroendocrine tumours, the recently proposed β-catenin, CD10, and PR were used, but not CD99, which is not specific for SPN.^19,26^ Instead, the CyclinD1, chromogranin and an additional, SOX11 were utilised (Table 1). The clue marker β-Catenin showed a consistent reaction between cytopathological (cell block) and resection samples. The differential diagnosis of pancreatoblastoma was dismissed with a negative CKAE1/AE3 reaction.

A diagnostic challenge arises because of the positive nuclear reaction to β-catenin, which is the marker of a somatic point mutation of the β-catenin gene (CCTNB), a driving mutation in more than 90% of SPN, but also present in most pancreatoblastomas and in 15% of pancreatic neuroendocrine neoplasms of higher stage (III/IV) but not in lower stage neoplasms.^19,20^ That is the reason why the adjunctive immunohistochemical staining to SOX11 is recommended, which is positive in SPNs and negative in normal pancreatic tissue and in neuroendocrine neoplasms. Additionally, it does not stain background cells and it does not have non-specific membranous or cytoplasmic staining as opposed to β-catenin.^24^

In general, the histopathological features make it difficult to predict the future behaviour of SPN, and radical resection is recommended in all cases, usually followed by an excellent prognosis, as in our cases.^20^ A more aggressive course is expected in tumours with high-grade malignant transformation, a higher number of mitoses and severe nuclear atypia.^20^ Vascular, lymphatic and perineural invasion or infiltrative growth into surrounding structures are not yet associated with a higher likelihood of relapse, as is also consistent with our second case.^20^ Overall, disease recurs in up to 15% of cases, most commonly in the local lymph nodes, liver and peritoneum, possibly years after the removal of the primary tumour and the metastasis.^28^ Compared to the adult population, the paediatric population with SPN has a better survival rate at all stages, despite the same treatment.^18,20^, ^23^ The 5-year survival rate of paediatric patients after completely resected SPN is 95%.^5^ In our institution both cases are without recurrence with a 31 and 50 months follow-up.

The sensitivity of EUS FNAB in diagnosing SPN in the adult population is estimated to be above 80%.^9^ In our institution, EUS-guided fine-needle aspiration biopsy (EUS FNAB) in diagnosing solid pseudopapillary neoplasms (SPN) in pediatric patients was so far accurate, based on two documented cases. In cases with necrotic samples, as in case 2, cytopathology specific techniques like filtration of cell sample to discard necrotic debris, and concentration are very helpful in detecting any individual viable cells, that can further undergo immunochemical stains. Unfortunately, necrosis and degenerated samples may also show a significant number of non-specific positive or false negative immunochemical reactions, making the diagnosis less reliable. The techniques that enable selection of viable diagnostic cells are not available in histology, which is yet another advantage of EUS FNAB.

The main drawback of the present study is the small sample size, connected to the rarity of the neoplasm, that could be avoided only by a multi-centric study.

## Conclusions

Solid pseudopapillary neoplasm (SPN) is a low-grade tumour that mainly affects young women and very rarely female pediatric patients. In the study, we documented two cases, successfully diagnosed by EUS FNAB, without any complications regarding the procedure. Main differential possibilities, neuroendocrine tumours and pancreatoblastomas, were both excluded by several immunochemical reactions, essential for the correct diagnosis. The caveat of one almost completely necrotic sample was overcome with an aid of filtration, the cytopathology specific technique, retrieving the sparse viable cells.

To sum it up, the diagnosis of SPN in the paediatric population requires a high level of suspicion and good collaboration of all specialties (paediatricians, gastroenterologists, radiologists, pathologists and surgeons) due to the wide spectrum of symptoms, the variety of radiological presentations and (cyto)pathological morphology. EUS FNAB proved to be a safe and efficient technique even at paediatric population.
